# The Doubtful Case of Diphtheria

**Published:** 1921-02-12

**Authors:** 


### EVERYDAY MEDICAL PROBLEMS.

### II.—The Doubtful Case of Diphtheria.

In these days of ready medical laboratory services it is not likely that the typical case of diphtheria, with its relatively simple bacteriological diagnosis, is going to be missed if the practitioner or nurse is reasonably observant and conscientious. But the very straightforwardness of taking a bacteriological swab, having it incubated for twelve or more hours, and awaiting the positive or negative result before continuing with a specific anti-toxin treatment, is liable to make us forget the number of curious anomalies and popular medical fallacies which arise in connection with the initial stages of this highly infectious disease. In a few words, however, it is possible to summarise only. We mention a point here, a section there, taken from a great clinical problem. Many of them are in practice more than often forgotten.

To start with, means for the bacteriological diagnosis may not be at hand. In that case all forms of throat affections in children should be regarded with suspicion, and precautionary measures of isolation, disinfection, and even anti-toxin administration adopted, if serious errors are to be avoided. Various apparently lighter forms of inflammation are often due to the diphtheria bacillus. It is by no means necessary to see the typical diphtheritic membrane before seriously considering the possibility. Also when due precautions *are* being taken it is well to remember that the diphtheria bacillus can remain alive upon infected articles for many months; scores of well-attested cases of transmission have arisen in this way. Pencils, sweets, etc., may be a potent factor in transmission. The throats of healthy contacts, nurses, for example, may pick up the bacilli, and develop into a most dangerous form of carriers. Remember, too, that infection does not necessarily occur upon the throat alone. It may affect the eyes as a conjunctivitis, the ears as an otitis, the surface of wounds, upon the genitals, as a fibrinous rhinitis in the nose, or even as an acute infection of the inner membranes and valves of the heart.

### The Import of a Membrane.

"Although the discovery of a tonsillar or faucial membrane" must in any case be regarded with suspicion, yet this appearance may also occur in scarlet fever, measles, Vincent's angina, whooping-cough, typhoid fever, or, in fact, any condition where bacterial, particularly streptococcal, infection of the throat happens to occur. The true diphtheritic membrane is given a classical description, but in practice it is often extraordinarily difficult to tell the true from the false. The early constitutional course of the disease, too, turns more than frequently into clinically atypical forms. Koplik's classification of these is probably the most satisfactory, and he describes them as—

(*a*) Those with no signs of membrane at all, but a simple catarrhal inflammation associated with a croupy cough. Bacteriological diagnosis alone can detect them, but such cases are of the greatest importance, since they may communicate the severe form of the disease to other children.

(*b*) Those in which the tonsils are covered with a pultaceous exudate, no typical membrane being formed.

(*c*) Cases showing a number of small punctate, difficultly recognisable patches of membrane.

(*d*) "Latent" diphtheria, often occurring in hospital practice, where the diphtheria bacilli already in the pharynx become active in young patients already subject to wasting conditions, and whose resistance has been gradually lowered thereby.

### Individual Susceptibility.

This question of individual resistance to the disease is important not only in the patient himself but more so among those he is likely to infect.

When attempting to localise the infection and adopt general precautionary measures in the home, school, or elsewhere, it is well to remember Osler's experience—that individual susceptibility is a very special factor, for not only do many of those exposed escape, but even those, too, in whose throats virulent bacilli lodge and grow. Probably about 70 per cent. of all persons have sufficient natural anti-toxin in their blood to protect them. Which of the contacts develop active disease is not, therefore, left to mere chance, and the possible value of a method detecting in a community those likely to be infected will be appreciated. Although not sufficiently recognised in this country, there is a specific test which can be carried out and should be more frequently used.

### The Schick Reaction.

By means of the so-called Schick test persons who have no natural immunity against diphtheria may be distinguished from those who have. A very small dose of diphtheria toxin is injected into the substance of the skin through a very fine needle. If positive, a reaction shows in twenty-four to forty-eight hours as an area of redness with slight infiltration about ½ inch or more in diameter, persisting painlessly for about a week. If the reaction is negative, nothing except a slight redness in the needle track is seen. Usually about one-fiftieth of the minimal lethal dose of toxin (for a guinea-pig) is injected and a negative reaction is taken as evidence in the person's system of sufficient anti-toxin to protect them against active diphtheria infection.

If a positive Schick test is obtained, or even if no test has been possible and we are dealing with an early possible contact, the question at once arises as to how we can provide an artificial immunity.

**The previous article appeared on Jan. 29, p. 405.**

**Everyday Medical Problems**—(*continued*).

In the first place, giving subcutaneous anti-toxin itself as a preventive measure is important. Its value is well known in children's hospitals, where it is frequently given as a routine prophylactic measure. To adults 1,000 units may be given, for older children 750 units, and to those up to two years of age, 500 units. In this way an undoubted immunity is obtained which lasts about three weeks. Recently mixtures of toxin and the equivalent amount of anti-toxin have been similarly inoculated in those persons having a positive Schick reaction. Three injections at weekly intervals produce an effect lasting for about two years, and the method is well worth a more extended trial in reducing the prevalence of the disease. The American cities already claim no little success in this direction.

Early Treatment.

In connection with early treatment it is needless to mention details in view of the predominant importance of anti-toxin administration. It is to be remembered in practice that anti-toxin may be injected subcutaneously, intramuscularly, or intravenously. The last is advisable in severe cases, and the intramuscular is always better than the simple subcutaneous route. But in this connection arises a frequently discussed difficulty. It is now a well-known fact that certain people are liable to anaphylactic or shock phenomena, collapse, etc., following upon the injection of the horse serum which conveys the anti-toxin. This may arise owing to previous administration of serum at some distant date or it may be a personal idiosyncrasy. Even if the occurrence be expected, however, the diphtheria patient must still be treated and the following methods are probably best adopted. A dose of two or three drops only of anti-toxin is first injected subcutaneously, and, if the person is susceptible, a reaction will occur within an hour, but probably it is better to wait for double this time before deciding if it is "safe." Should the patient be found sensitive and the need of anti-toxin be great, then small doses (2 to 4 c.c.) should be given at hourly intervals until a total requisite amount has been injected. Children, in whom diphtheria is much more frequent than in adults, seem to be far less liable to exhibit this sensitiveness to serum, and for this fact both the parents and the doctor may be duly thankful.

